# DRBD-YOLOv8: A Lightweight and Efficient Anti-UAV Detection Model

**DOI:** 10.3390/s24227148

**Published:** 2024-11-07

**Authors:** Panpan Jiang, Xiaohua Yang, Yaping Wan, Tiejun Zeng, Mingxing Nie, Zhenghai Liu

**Affiliations:** 1 School of Nuclear Science and Technology, University of South China, Hengyang 421001, China; 2 Intelligent Nuclear Security Technology Laboratory, Hengyang 421001, China; 3 School of Civil Engineering, University of South China, Hengyang 421001, China; 4 School of Computer, University of South China, Hengyang 421001, China

**Keywords:** anti-UAV detection, lightweight, YOLOv8n, BiFPN, loss function, edge-computing devices

## Abstract

Interest in anti-UAV detection systems has increased due to growing concerns about the security and privacy issues associated with unmanned aerial vehicles (UAVs). Achieving real-time detection with high accuracy, while accommodating the limited resources of edge-computing devices poses a significant challenge for anti-UAV detection. Existing deep learning-based models for anti-UAV detection often cannot balance accuracy, processing speed, model size, and computational efficiency. To address these limitations, a lightweight and efficient anti-UAV detection model, DRBD-YOLOv8, is proposed in this paper. The model integrates several innovations, including the application of a Re-parameterization Cross-Stage Efficient Layered Attention Network (RCELAN) and a Bidirectional Feature Pyramid Network (BiFPN), to enhance feature processing capabilities while maintaining a lightweight design. Furthermore, DN-ShapeIoU, a novel loss function, has been established to enhance detection accuracy, and depthwise separable convolutions have been included to decrease computational complexity. The experimental results showed that the proposed model outperformed YOLOV8n in terms of mAP50, mAP95, precision, and FPS while reducing GFLOPs and parameter count. The DRBD-YOLOv8 model is almost half the size of the YOLOv8n model, measuring 3.25 M. Its small size, fast speed, and high accuracy combine to provide a lightweight, accurate device that is excellent for real-time anti-UAV detection on edge-computing devices.

## 1. Introduction

As electrical and communication technology has advanced, drones have grown significantly and are now employed extensively in many different sectors [1,2,3,4]. Despite their benefits, drones also bring high risks, particularly regarding safety and privacy. In critical areas like military sites, nuclear power plants, airports, and government buildings, the malicious use of drones can lead to serious consequences [5]. Incidents involving unapproved UAV flights and terrorist assaults using drones have also increased [6,7,8]. To effectively address these risks, and to enforce policies and regulations [9,10] that can limit drone production and usage, it is essential to employ advanced technical solutions to defend against UAV threats.

Real-time detection and identification are critical aspects of anti-UAV technology, serving as the foundation for implementing effective countermeasures. Current mainstream drone detection technologies primarily rely on a variety of sensors, including infrared, radar, photoelectric, acoustic, and radio frequency sensors [11,12]. The most widely used method for low-altitude UAV detection is computer vision-based target detection. Traditional computer vision detection techniques generally use a sliding window method to count potential frames in the image and extract important feature information before categorizing and identifying the UAV [13,14]. However, these traditional methods are often computationally intensive and time-consuming and demonstrate poor feature extraction capabilities and low accuracy in anti-UAV detections. In recent years, deep learning-based computer vision technologies have advanced rapidly, and numerous highly effective object detection algorithms have emerged [15,16,17,18,19]. The YOLO series is particularly outstanding among these algorithms. Numerous fields have found uses for the YOLO family including intelligent security [20], autonomous driving [21], and medical image analysis [22]. They also present new opportunities for real-time UAV detection and recognition.

While anti-UAV detection duties are becoming more and more complicated, they also face a variety of unique problems. Anti-UAV detection models require high accuracy and real-time processing to successfully counter UAV threats, a criterion that many object identification algorithms find difficult to meet. Furthermore, these detection models are often deployed on outdoor edge-computing devices, which have limited hardware resources, making it impractical to deploy large models. Thus, the lightweight design for drone detection models is essential. Moreover, motion blur and occlusion are unavoidable for small, moving targets like drones, which can impair image quality and reduce detection precision.

To address these challenges, substantial research has been dedicated to developing UAV detection algorithms based on the YOLO series. For instance, Behera et al. [23] conducted extensive comparisons and ultimately adopted YOLOv3 for UAV detection and classification, achieving a best detection accuracy of 74%. Phung et al. [24] combined YOLOV4 and the CSRT tracking algorithm to detect and track UAVs, obtaining a detection accuracy of 81% and a frame rate of approximately 10 FPS. Singha et al. [25] used YOLOv4 to detect drones in the video and obtained a mAP of 74.36%. The performance of UAV detection using only the YOLO algorithm is relatively low. The main reason for this is that the YOLO series has several drawbacks, such as its insensitivity to small targets, slow processing speed on edge devices, and particularly, its inability to strike the perfect balance between accuracy and model efficiency. Therefore, researchers have implemented several enhancements to the YOLO algorithm to improve its detection effectiveness. To further refine the detection of small drones in complex environments, Bo et al. [26] introduced the YOLOv7-GS model, which incorporates the SPPCSPC-SR module with a simple attention mechanism to focus on regions with small objects, reducing information loss and minimizing missed detections. Tian et al. [27] developed the MD-YOLO algorithm, which integrates a multi-scale feature fusion module and an adaptive attention module to improve the sensitivity of small targets. Wu et al. [28] introduced a small object detection layer to the original YOLOv8n, significantly improving the model’s detection ability to capture small targets’ information. These three techniques improved detection accuracy, albeit at the cost of higher computational and parameter loads. For example, the GFLOPS of YOLOv7-GS is 15.5, which exceeds that of the original YOLOv7-tiny (13.2). Moreover, the model size for YOLOv7-GS is 23.2 MB, nearly doubling that of YOLOv7 (12.3 MB). To optimize the performance of YOLO on edge-computing devices, various lightweight enhancements have been proposed. Liu et al. [29] designed an efficient feature extraction module for the YOLO backbone, which extracts meaningful features while reducing model size and computational costs. Huang et al. [30] improved YOLOv8 by integrating ghost convolution to reduce model parameters, creating a compact model of just 4.23 MB. To capture more features and improve drone recognition accuracy, the model was updated with three effective multi-scale attention modules. However, this approach resulted in longer processing times and higher computational expenses. Zhou et al. [31] improved YOLOv4 by introducing VDTNet, which uses model compression to reduce its size to 3.9 MB. A spatial attention module compensates for accuracy by displaying longer latencies and moderate speeds. Liu et al. [32] refined YOLOv4, applying pruning to minimize parameter count and small object enhancement to offset precision loss. However, the final model size remained at 15.1 MB, which is still too large for an edge device.

In summary, most current anti-UAV detection methods based on the YOLO family struggle to strike an optimal balance between accuracy, speed, and computational efficiency. To overcome these limitations, this study presents a new drone detection model, DRBD-YOLOv8. This model has a series of improvements based on YOLOv8 and demonstrates fast computational speeds, high detection accuracy, low computational complexity, and minimal memory usage. The key contributions of this study include the following:

- A high-performance and lightweight anti-UAV detection model was developed. The model integrates depthwise separable convolutions into the backbone for a more lightweight design. To reconfigure the neck network, a novel fusion module named RCELAN was presented, which strikes a balance between lightness, fast inference, and high precision. Moreover, a BiFPN architecture was introduced in the neck to facilitate multi-scale feature fusion, improving the model’s ability to detect small objects while significantly reducing complexity and the number of parameters.
- A comprehensive UAV dataset was created, featuring images of UAVs in various environments, such as urban areas, mountains, forests, and highways. This dataset reflects the complex real-world scenarios and provides rich data for training the detection model.
- A new loss function called DN-ShapeIoU was proposed, which considers both the shape and scale of bounding boxes and employs a dynamic nonlinear gradient strategy based on sample quality during loss calculation. By reducing the detrimental effects of poor-quality images, DN-ShapeIoU raises detection accuracy overall and enhances bounding box regression.

The rest of this paper is organized as follows: Section 2 reviews related work on anti-UAV detection technologies powered by deep learning. Section 3 introduces the DRBD-YOLOv8 anti-UAV detection model in detail. Section 4 presents a series of comparative experiments, along with an extensive analysis of the experimental results. Section 5 summarizes the study and presents a perspective on future work.

## 2. Materials and Methods

This paper proposes a high-performance and lightweight anti-UAV detection model called DRBD-YOLOv8. The adoption of current models on edge-computing devices has been impeded by their huge bulk and slow speed. These restrictions are addressed by this model.

**Figure 1:**
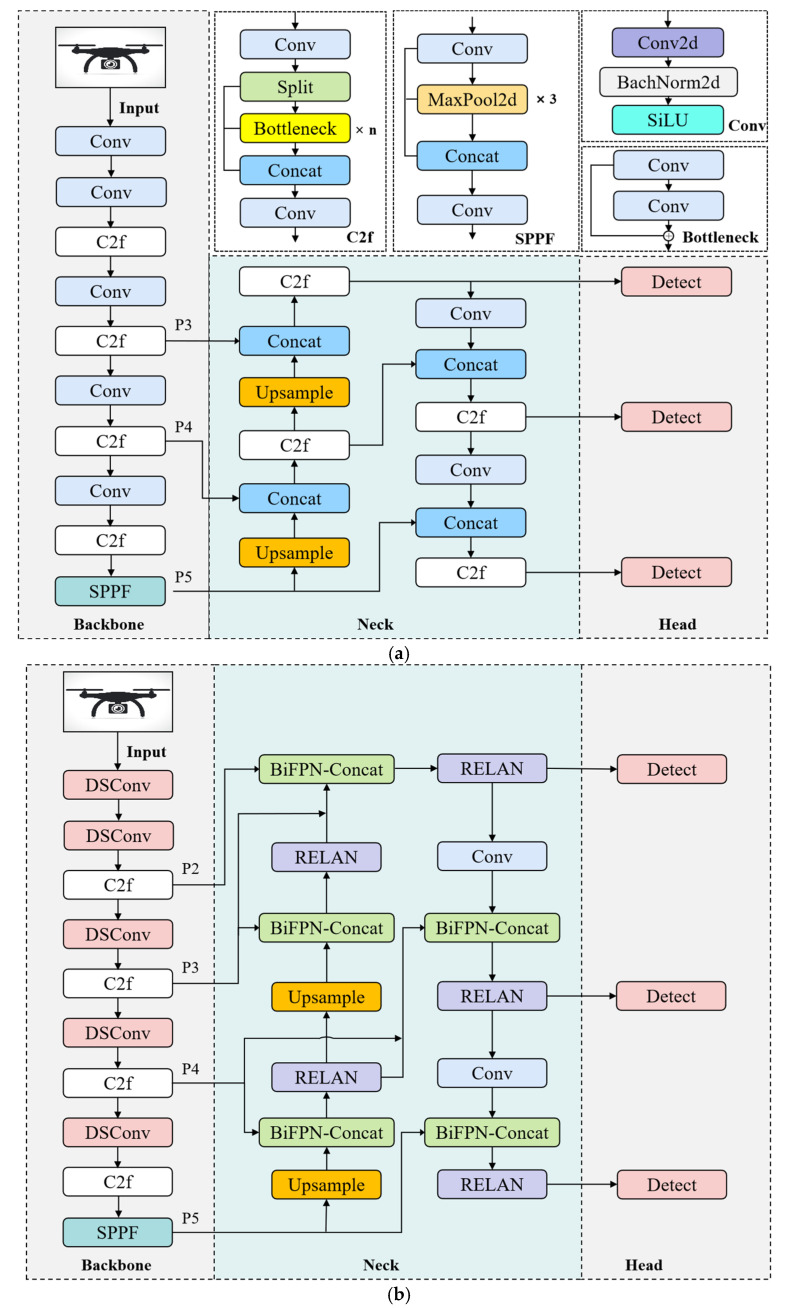
Structure of (**a**) YOLOv8 and (**b**) DRBD-YOLOv8. The structure of the Conv, C2f, and SPPF modules in (**b**) is the same as that of (**a**).

Compared to YOLOv8, the DRBD-YOLOv8 model has several significant improvements (Figure 1). First, a more effective and compact model is produced by substituting depthwise separable convolutions for the standard convolution in the backbone. Secondly, the proposed RCELAN module is introduced in place of the original C2f module in the neck. This RCELAN module improves multi-level feature extraction by integrating feature map segmentation with gradient path optimization, all while reducing the computational load and number of parameters. A significant addition is the integration of RepConv [33], which employs structural re-parameterization to improve inference speed and operational efficiency. Thirdly, recognizing the small size of drones, the BiFPN architecture is implemented to enhance the fusion of multi-scale information and improve the model’s ability to identify small targets. Lastly, to address the negative impact of low-quality training samples, the DN-ShapeIoU loss function is introduced. This loss function prioritizes the shape and size of bounding boxes and applies a dynamic nonlinear gradient strategy based on sample quality to refine the model’s bounding box regression and detection accuracy.

### 2.1. Depthwise Separable Convolutions

**Figure 2:**
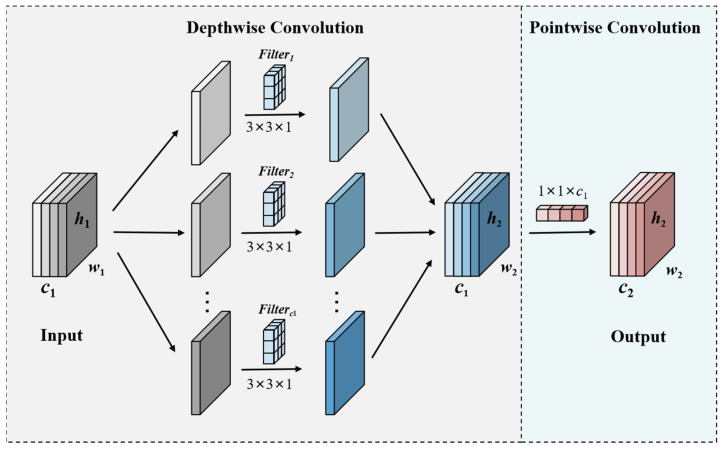
The calculation process of a depthwise separable convolution.

Depthwise separable convolution has several advantages over standard convolution, the main one being its capacity to significantly decrease model parameters and computational load without sacrificing network performance. Depthwise separable convolutions have become widely used in several lightweight network topologies because of their enhanced efficiency [34,35]. In this study, the backbone of YOLOv8n is replaced with depthwise separable convolutions, allowing for a more simplified and lightweight model design. The two components of depthwise separable convolutions are pointwise convolution and depthwise convolution. The former extracts spatial features across individual channels, while the pointwise convolution, applied to the output of the depthwise stage, merges spatial positional features to enhance the richness of feature representation. The computation of depthwise separable convolutions is shown in Figure 2. Given an input feature map with dimensions of *h*_1_ × *w*_1_ × *c*_1_, depthwise convolution is applied using C_1_ groups of *k* × *k* × 1 kernels (where *k* = 3 in this case), producing C_1_ feature maps for each channel. These maps are then subjected to pointwise convolution, which uses C_2_ groups of 1 × 1 × *c*_1_ kernels, yielding the final output feature map with dimensions *h*_2_ × *w*_2_ × *c*_2_.

Given that *P*_1_ and *F*_1_ represent the number of parameters and computational cost for a depthwise separable convolution, the following formulas can be obtained:

(1) $$P_{1}=c_{1}\times k\times k\times 1+c_{2}\times 1\times 1\times c_{1}$$

(2) $$F_{1}=c_{1}\times h_{2}\times w_{2}\times k\times k\times 1+c_{2}\times h_{2}\times w_{2}\times 1\times 1\times c_{1}$$

For the same input feature map, obtaining an output feature map of dimensions *h*_2_ × *w*_2_ × *c*_2_ using standard convolution involves the parameter count and computational cost represented by *P*_2_ and *F*_2_, respectively. These are expressed as follows:

(3) $$P_{2}=c_{2}\times c_{1}\times k\times k$$

(4) $$F_{2}=c_{2}\times h_{2}\times w_{2}\times c_{1}\times k\times k$$

where *k* = 3. By comparing *P*_1_ with *P*_2_ and *F*_1_ with *F*_2_, the following expression can be derived:

(5) $$\frac{P_{1}}{P_{2}}=\;\frac{1}{c_{2}}+\frac{1}{9}$$

(6) $$\frac{F_{1}}{F_{2}}=\frac{1}{c_{2}}+\frac{1}{9}$$

Equations (5) and (6) show that the computational cost and parameter count of the depthwise separable convolution are significantly reduced to around one-ninth of those of the standard convolution. This significant reduction is highly advantageous for creating lightweight models, particularly in UAV detection systems.

### 2.2. RCELAN Module

**Figure 3:**
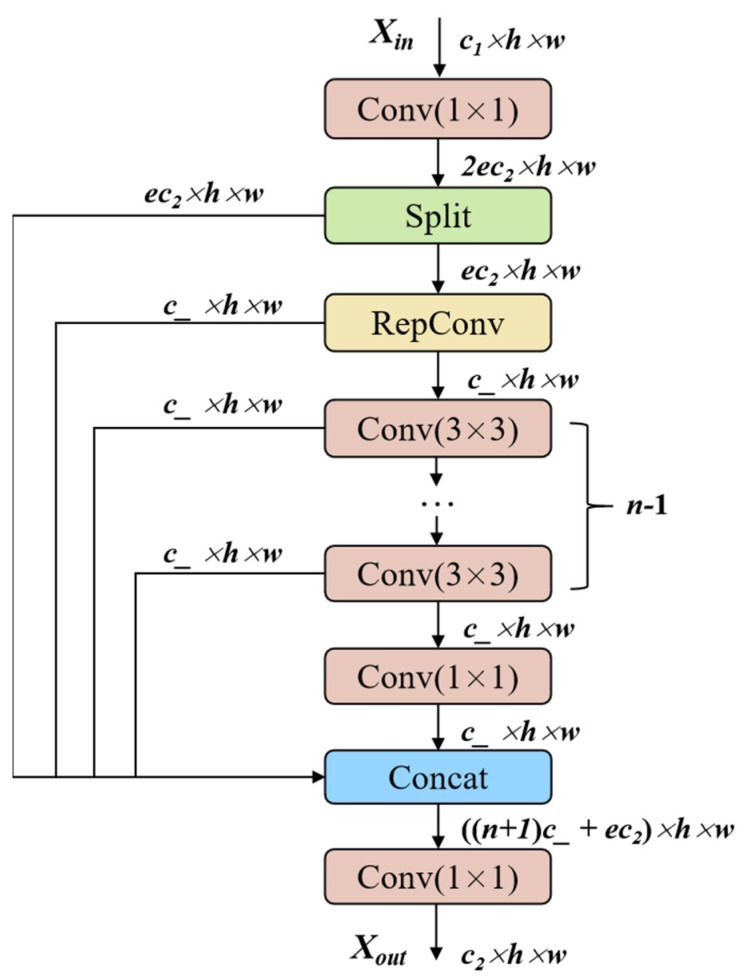
The architecture of the RCELAN module.

The RCELAN module was developed with inspiration from cutting-edge networks like CSPNet [36] and ELAN [37]. It strikes an ideal mix between inference speed, detection accuracy, and lightweight architecture. To alter the channels, the input feature maps are first subjected to a one-dimensional convolution, as shown in Figure 3. They are then split into two parts: one part is retained as the original feature representation, while the other part proceeds through several convolution operations along the main branch. The parameter e represents the expansion ratio, which adjusts the number of channels in the hidden layers, thereby controlling both the network’s capacity and computational complexity. Typically, *e* is set between 0 and 1, where a higher value increases the channels, enabling richer feature capture but also increasing computational cost. On the other hand, a lower *e* value reduces complexity but may limit the model’s capability to learn features. The parameter *s* acts as a scaling factor that adjusts the channel dimensions of the intermediate layers, and its value can be any positive number. The model size can be adjusted by modifying it to meet the needs of various networks. In this study, both *e* and *s* are set to 0.5, where *c_* is the intermediate channel dimension and *c_ = s × e × c*_2_.

The bottleneck structure is eliminated by the RCELAN module, in contrast to the C2f structure of the original YOLOv8. To improve feature extraction and gradient propagation, the RCELAN module integrates RepConv into the main branch to offset the performance loss brought about by the removal of residual blocks in the bottleneck. RepConv executes operations in parallel using 1 × 1 and 3 × 3 convolutions, improving multi-scale feature extraction. The main branch then applies 3 × 3 convolutions for local feature extraction, followed by additional layers of 3 × 3 convolutions to capture more complex and deeper features. Finally, a 1 × 1 convolution captures broader semantic information. The RCELAN module merges the unprocessed features from the split operation with those generated by the main branch, resulting in multi-level feature fusion. A final 1 × 1 convolution adjusts the output channels to produce the final feature map.

**Figure 4:**
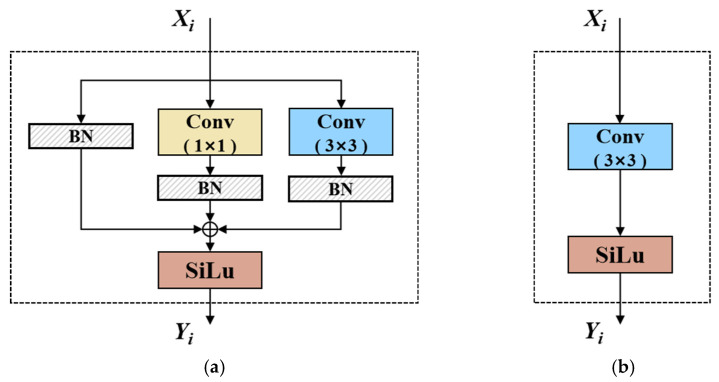
The architecture of RepConv: (**a**) training and (**b**) inference.

RepConv uses a multi-branch convolutional layer in training, as seen in Figure 4. This setup includes a parallel 3 × 3 convolutional layer, a 1 × 1 convolutional layer, and an identity branch. During inference, the parameters from each branch are extracted and merged through re-parameterization techniques, resulting in a single set of equivalent weights and biases, effectively condensing multiple layers into one. The formula for batch normalization (*BN*) is as follows:

(7) $$X_{b}=W_{BN}*x_{b}+b_{BN}$$

(8) $$W_{BN}=\frac{\gamma }{\sqrt{\sigma^{2}+\varepsilon }}$$

(9) $$b_{BN}=\beta -\frac{\gamma *\mu }{\sqrt{\sigma^{2}+\varepsilon }}$$

where *μ* and *σ* represent the mean and standard deviation of the *BN* channel, respectively; *γ* indicates the learned scale factor; β signifies the learned bias term; *x_b_* denotes the input to the *BN* layer; *X_b_* represents the output after *B_N_*; *W_BN_* indicates the equivalent weight; and *b_BN_* denotes the equivalent bias. The fusion formula of the convolution and normalization layers is depicted as follows:

(10) $$X_{cb}=W_{CB}*x_{c}+b_{CB}$$

(11) $$W_{CB}=W_{BN}*W_{C}$$

(12) $$b_{CB}=W_{BN}*b_{C}+b_{BN}$$

where *x_c_* denotes the convolution input; *W_c_* and *b_c_* represent the weight and bias of the convolution, respectively; *W_CB_* and *b_CB_* represent the fused weight and bias; and *X_cb_* indicates the output after fusion. During inference, the three branches consisting of the 3 × 3 and 1 × 1 convolution layers and identity branch are combined into a single branch containing only the 3 × 3 convolution. The final output, *Y_i_*, can be expressed as follows:

(13) $$Y_{i}=W_{fuse}*X_{i}+b_{fuse}$$

(14) $$W_{fuse}=W_{I}^{0\times 0}+W_{fuse}^{1\times 1}\;{+\;W}_{fuse}^{3\times 3}$$

(15) $$b_{fuse}=b_{I}^{0\times 0}+b_{fuse}^{1\times 1}+b_{fuse}^{3\times 3}$$

The final output Y_i_ can be expressed as Equations (13)–(15), where *W_fuse_* and *b_fuse_* denote the parameters of the final convolution layer. The terms $W_{I}^{0\times 0}$, $W_{fuse}^{1\times 1}$, and $W_{fuse}^{3\times 3}$ represent the weights obtained from the fusion of convolution and *BN* layer for each branch, corresponding to the identity, 1 × 1, and 3 × 3 convolutional layers, respectively. Similarly, $b_{I}^{0\times 0}$, $b_{fuse}^{1\times 1}$, and $b_{fuse}^{3\times 3}$ represent the biases derived from the same fusion process for each branch.

The re-parameterization technique significantly reduces model complexity and memory usage, allowing for a more compact and efficient design. Furthermore, during inference, the decrease in layer transitions and parameter processes leads to enhanced computational efficiency, which expedites the speed of inference. Significantly, the learning capacity of the model is unaffected because the fusion operations are mathematically comparable.

### 2.3. BiFPN Incorporating a Small Target Detection Layer

**Figure 5:**
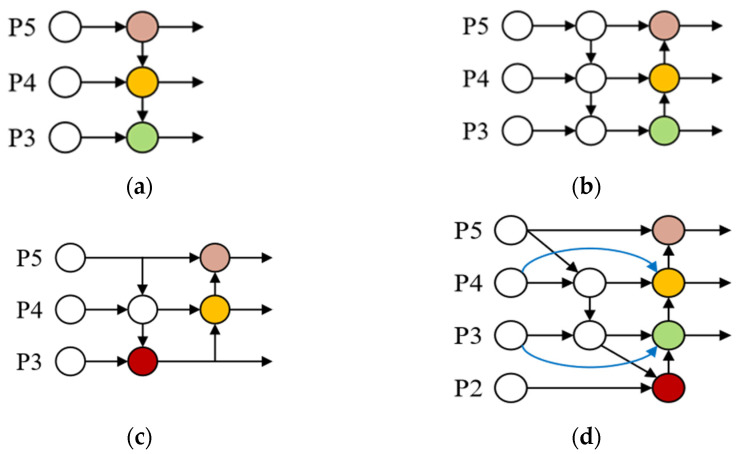
Feature extraction network design: (**a**) FPN, (**b**) PAN, (**c**) PAFPN, and (**d**) BiFPN.

The feature extraction layer of YOLOv3 employs the FPN architecture [38], which establishes a top-down pathway with lateral connections to fuse features from different layers. This architecture improves the model’s ability to capture rich features at different scales. However, due to its unidirectional information flow, the detection accuracy remains limited, especially in handling complex multi-scale object features. PAN [39] improves FPN by introducing a bottom-up pathway, which enhances the flow of information between different feature levels. PAN combines high-level semantic features from the top layers with low-level positional features, thus allowing the prediction feature map to contain both precise spatial and abstract semantic information. Figure 5a,b illustrate the structures of FPN and PAN, respectively. Figure 5c presents the PAFPN structure employed by YOLOv5 and YOLOv8 for multi-layer feature fusion. By removing nodes that make insignificant contributions to feature fusion, PAFPN streamlines the design and combines the best aspects of PAN and FPN. This simplification promotes the transfer and integration of abstract semantic details with concrete spatial details, ultimately enhancing the performance of object detection tasks. However, in PAFPN, feature information from different scales is combined using concatenation without distinguishing the varying significance of these features.

In this study, the BiFPN structure is employed for feature fusion. As shown in Figure 5d, BiFPN highlights important features by assigning weights to each input during the fusion process at different levels. The removal of low-contribution nodes and the establishment of horizontal connections improve the fusion and flow of multi-scale data in the network. To improve detection performance for small objects, the approach strategically incorporates low-level features from the P2 layer into high-level information. In contrast to standard procedures, the feature fusion of the P2 layer is still the major focus, and no more detection heads are added. The computational load of the network would be greatly increased by adding more detecting heads, making it more difficult to maintain a lightweight architecture.

The BiFPN architecture makes the network more capable of identifying and locating small objects by fully integrating P2 layer elements. Accurate UAV recognition depends on reducing false positive and false negative rates, which is because this improvement enhances the detection capabilities for multi-scale objects.

### 2.4. DN-ShapeIoU

Bounding box regression loss functions are designed to measure the discrepancy between predicted and ground truth bounding boxes. Typically, these loss functions are based on Intersection over Union (IoU), which measures the overlap between the predicted and actual bounding boxes [40]. However, anchor box regression faces difficulties when dealing with small, moving targets such as drones due to the unpredictability of bounding box shape and scale. Moreover, UAV datasets often include blurred, low-resolution, and occluded samples, reflecting real-world conditions that complicate UAV detection. The YOLOv8 loss function, which uses the CIOU, has problems handling these problems well. To overcome this limitation, the method proposed in this study introduces a novel loss function named *DN-ShapeIoU*.

The *Shape-IoU* loss function [40] thoroughly considers the shape and size of the bounding box to enhance regression performance. It introduces a shape weighting factor that dynamically adjusts the loss contributions along the horizontal and vertical axes. To further improve the accuracy of the regression, the function also includes factors for shape distance and angle loss. The mathematical expression for *Shape-IoU* is given by the following equation:

(16) $$L_{ShapeIOU}=1-IoU+D+0.5\Omega$$

(17) $$IoU=\frac{\left|B\cap B_{gt}\right|}{\left|B\cup B_{gt}\right|}$$

(18) $$D=\frac{hh\times {\left(x_{c}-x_{gt}\right)}^{2}+ww\times {\left(y_{c}-y_{gt}\right)}^{2}}{c^{2}}$$

(19) $$\Omega =\sum_{t=w,h}{\left(1-e^{-w_{t}}\right)}^{4}$$

where *D* represents the shape distance, *Ω* refers to the angle loss, *B* denotes the area of the predicted bounding box, and *B_gt_* signifies the area of the ground truth bounding box.

**Figure 6:**
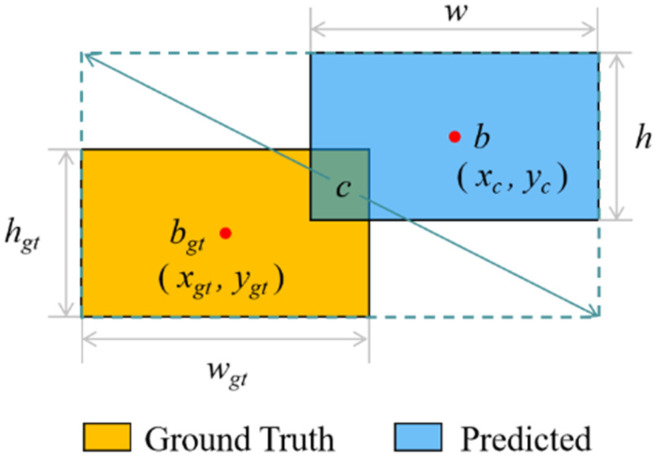
Schematic diagram of IoU.

As illustrated in Figure 6, *b_gt_* is the center point of *B_gt_* with coordinates (*x_gt_*, *y_gt_*), while *b* is the center of *B*, with coordinates (*x_c_*, *y_c_*). The variable *c* refers to the diagonal distance of the minimum enclosing bounding box that covers both *B* and *B_gt_*. The horizontal and vertical weight coefficients are represented by *ww* and *hh*, respectively. The related expressions are presented in Equations (20)–(23). The size of the target object in the dataset is related to the scale factor, which is represented by the symbol scale. Moreover, *w* and *h* represent the width and height of the predicted bounding box *B*, while *w_gt_* and *h_gt_* indicate the width and height of the ground truth bounding box *B_gt_*.

(20) $$ww=\frac{2\times {\left(w_{gt}\right)}^{scale}}{{\left(w_{gt}\right)}^{scale}+{\left(h_{gt}\right)}^{scale}}$$

(21) $$hh=\frac{2\times {\left(h_{gt}\right)}^{scale}}{{\left(w_{gt}\right)}^{scale}+{\left(h_{gt}\right)}^{scale}}$$

(22) $$w_{w}=hh\times \frac{\left|w-w_{gt}\right|}{\max\left(w,w_{gt}\right)}$$

(23) $$w_{h}=ww\times \frac{\left|h-h_{gt}\right|}{\max\left(h,h_{gt}\right)}$$

Although Shape-IoU [40] improved bounding box regression accuracy, it is not able to distinguish between gradient contributions depending on the various qualities of training data. During training, low-quality samples can generate misleading gradients, which may result in abnormal gradient gains and disrupt the model’s ability to learn accurate features. To tackle this problem, this work presents an enhanced loss function called DN-ShapeIoU, which is based on Shape-IoU and was motivated by the work [41]. It performs this by reducing the negative impact of poor-quality samples on model training, thus increasing the generalization capacity of the model. The proposed DN-ShapeIoU incorporates an adaptive nonlinear focusing mechanism, which dynamically adjusts the gradient gain according to the quality of the samples, thereby optimizing the gradient flow during training. The mathematical formulation of DN-ShapeIoU is shown in Equation (24), where *γ* denotes the adaptive gradient gain.

(24) $$L_{DN-ShapeIOU}=\gamma \times L_{ShapeIOU}$$

(25) $$\gamma =\frac{\tau }{\epsilon \;\alpha^{\tau -\epsilon }}$$

(26) $$\tau =\frac{L_{ShapeIOU}^{*}}{A_{L_{ShapeIOU}}}\;\in \left[0,+\infty \right]$$

The formulation of *γ* is provided in Equation (25), where *α* and *ϵ* represent hyperparameters, and *τ* represents the outlier degree, as defined in Equation (26). In this context, L*_shapeIoU_ refers to the non-gradient version of the current *L_shapeIoU_*, and *A_LshapeIoU_* is the exponential moving average of *L_shapeIoU_* with a momentum factor m. The value of *γ* is dynamically adjusted according to the quality of the anchor box during training. Specifically, for high-quality anchor boxes with a low outlier degree, the mechanism assigns a small value for *γ* to limit the update magnitude, avoiding over-adjustments for these well-defined samples. To prevent the propagation of hazardous gradients, a similarly low gradient gain is used for low-quality anchor boxes that show high outlier degrees. This adaptive nonlinear mechanism assigns gradient gains based on the outlier degree rather than the IoU values, facilitating more intelligent gradient distribution during training.

To sum up, the DN-ShapeIoU loss function includes an adaptive nonlinear focusing method that modifies the gradient gain according to sample outlier degrees in addition to addressing the variations in bounding box shape and scale among UAV datasets. By reducing the disruptive impact of substandard UAV samples, this technique significantly improves the detection accuracy and overall robustness of the model.

## 3. Experiment and Analysis

This section outlines our experimental approach, starting with the experimental setup, including the dataset, experimental environment, parameter settings, and evaluation metrics; then, four comparative experiments are conducted to evaluate the innovations in this paper, which are the comparison experiments with depthwise separable convolutions, RCELAN, the BiFPN structure, and DN-ShapeIoU, with ablation experiments to prove the contributions of their individual effects. Moreover, the DRBD-YOLOv8 model is further compared with other mainstream YOLO series algorithms on our drone dataset and quantitative comparisons are carried out on the public dataset to verify the model’s generalization ability and versatility. Finally, the detection results are visualized to illustrate the effectiveness of the proposed enhancements.

### 3.1. Dataset

**Figure 7:**
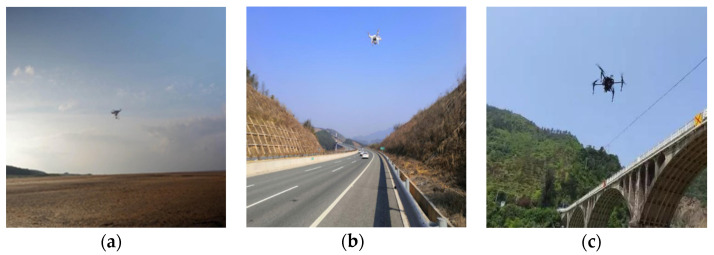

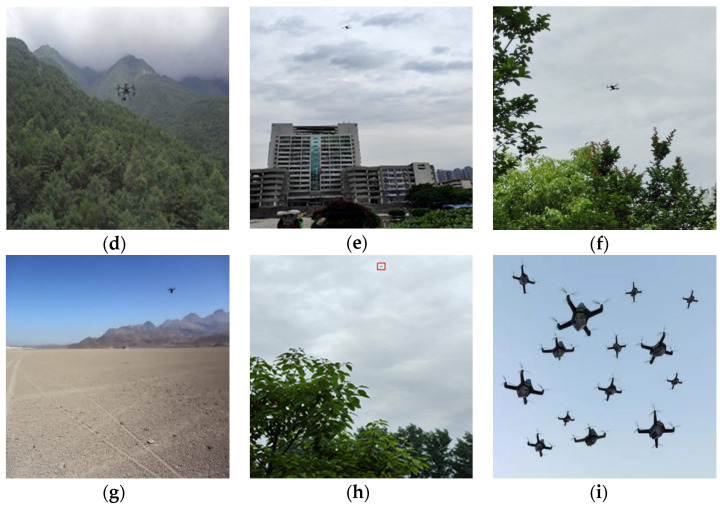
Dataset samples, with drones flying in different scenarios: (**a**) over the prairie, (**b**) in the expressway, (**c**) near the bridge, (**d**) in the mountains, (**e**) over the city, (**f**) through the woods, (**g**) over the desert, (**h**) in the clouds far away, where the drone is in the red box, and (**i**) scenes with multiple drones.

A comprehensive dataset was constructed for drone detection. This dataset encompasses 14,509 images of drones captured in various environments, such as urban areas, mountainous regions, forests, bridges, and deserts. The dataset has been randomly divided into three parts: a train set, a validation set, and a test set, with a ratio of 6:2:2. It includes 15 different types of drones, featuring both single-drone and multi-drone scenarios. The images were sourced from publicly available VAU videos and images, as well as experimental captures. Figure 7 provides a selection of drone images from this dataset, demonstrating its diversity and its close alignment with real-world conditions. This dataset offers a rich learning resource for training and evaluating the model.

### 3.2. Parameter Settings

All experiments were carried out using an i9-12900KF CPU (Intel, Shanghai, China) running at 3.2 GHz, and 32 GB of RAM, with a single NVIDIA GeForce RTX 3060 GPU (NVIDIA, Shanghai, China) having 12 GB of dedicated memory. The software employed was the PyTorch 2.3.0 framework, paired with torchvision 0.18.0, alongside Python 3.8.0 and CUDA version 12.1 for GPU acceleration. The key training parameters are listed in Table 1.

To comprehensively evaluate the performance of the proposed model, several evaluation metrics were employed: precision (P), mean average precision (mAP), GFLOPs, recall (R), parameters (Param), model size, and FPS. The average precision (AP) is calculated as the area under the P-R curve. The metric mAP50 refers to the mean AP across all classes at a 50% IoU threshold, while mAP95 follows the same definition but averages the AP values at various IoU thresholds up to 95%. The detailed calculations for AP and mAP are provided in Equations (27) and (28), respectively, where *n* represents the number of classes. FPS reflects the total time spent on data preprocessing (*T_pre_*), model inference (*T_infer_*), and postprocessing (*T_post_*), as shown in Equation (29).

(27) $$AP=\int_{0}^{1}P\left(R\right)dR\;$$

(28) $$mAP=\frac{1}{n}\sum_{i=1}^{n}AP_{i}$$

(29) $$FPS=\frac{1}{T_{pre}+T_{infer}+T_{post}}$$

### 3.3. Experimental Comparison and Analysis

#### 3.3.1. Comparison Effect of Different Convolutions

Comparative studies were carried out by including multiple commonly used lightweight convolutions into the YOLOv8n model to validate the efficacy of depthwise separable convolutions. In these experiments, the standard convolution (Conv) in the YOLOv8n backbone was replaced with alternative types, such as depthwise convolution (DWConv), ghost convolution (GhostConv), pointwise convolution (PWConv), group convolution (GConv), and depthwise separable convolution (DSConv). Table 2 provides a summary of the particular results of these experiments and illustrates the contribution of each convolutional variant. It is important to note that all comparison tests were carried out in the same conditions, and the results shown in this and all subsequent experiments are based on evaluations using the test set.

Table 2 shows that a similar decrease in GFLOPs and parameters is obtained when several lightweight convolution approaches are substituted for the standard convolution in the backbone of YOLOv8n. However, the performance improvements vary among these techniques. For example, mAP50 and mAP95 decrease with GhostConv and PWConv, but mAP50 and mAP95 slightly increase with DWConv and GConv. Among the approaches that have been studied, DSConv has demonstrated the greatest improvement in mAP50, mAP95, and GFLOPs. Although the P, R, and parameter metrics for DSConv are slightly below the optimal levels, it achieves reductions of 0.9 G in GFLOPs and 0.35 M in parameters, approximately an 11% decrease from the original values. This highlights the effectiveness of DSConv in reducing model complexity while capturing more detailed features, thus making it the most advantageous for lightweight models.

#### 3.3.2. Comparison Experiments with RCELAN Module

(1) Comparison effect of different feature extraction modules

Comparative experiments were carried out against the C3 module from YOLOv5 [42], the ELAN module from YOLOv7 [43], the C2f module from YOLOv8 [44], and the RepNCSPELAN4 module from YOLOv9 [45] to evaluate the proposed performance of the RCELAN module. For these experiments, the input and output feature map dimensions were set to (1, 1, 128, 128). It is evident from Table 3 that the RCELAN module demonstrated the lowest parameter count and computational cost while achieving the highest FPS (frames per second) value when inputting the same feature maps. In conclusion, the RCELAN module performed well in terms of processing speed, detection accuracy, and lightweight design.

(2) Comparison effect of the RCELAN module at different positions

In this study, the RCELAN module was developed to substitute the C2f module in YOLOv8, to improve the understanding and fusion of feature maps. Since YOLOv8 includes several C2f modules, the effect of changing these modules at different places was investigated using four different experimental configurations: The first experiment involved no substitutions. The second experiment replaced only the C2f modules within the YOLOv8n backbone. The third experiment focused on substituting the C2f modules within the YOLOv8n neck. The fourth experiment replaced all C2f modules in YOLOv8n with RCELAN modules.

Table 4 demonstrates that a significant decrease in GFLOPs and parameters resulted from substituting all the C2f modules with RCELAN modules. However, comparing this complete replacement to the original YOLOv8n, the values of mAP50, mAP95, P, and R were lower. With the greatest mAP50, mAP95, and precision values among the studies, the third group performed the best, involving the replacement of solely the C2f modules in the neck.

Compared to the original YOLOv8n, the third set of experiments achieved a 13% reduction in parameters and an approximate 10% decrease in GFLOPs while showing a minor improvement in both mAP50 and mAP95. This indicates that replacing the C2f modules with RCELAN modules in the neck provides a balanced trade-off between detection performance and the lightweight design of the model.

#### 3.3.3. Comparison Effect of BiFPN Architecture

A set of experiments was carried out to assess the improved information fusion capabilities brought about by the BiFPN architecture, which integrates features across several scales in the neck network. These experiments compared the standard YOLOv8n model with its counterpart incorporating the BiFPN structure. The results highlight the improvements gained through the enhanced feature fusion and illustrate the value of BiFPN in strengthening the model’s detection performance.

It is evident from Table 5 that the BiFPN architecture reduces complexity and computational demands while significantly improving feature information fusion in the neck part of the model. Compared to YOLOv8n, the model with BiFPN shows improvements in mAP50, mAP95, R, and P when compared to YOLOv8n. This enhancement primarily originates from the ability of BiFPN to effectively merge multi-scale information, particularly through the integration of feature data from the small target layer P2. Moreover, the use of BiFPN resulted in a 12.3% reduction in GFLOPs, a 33.9% decrease in parameter count, and a 32.3% reduction in model size, highlighting its effectiveness in creating a more lightweight model suitable for edge device deployment.

#### 3.3.4. Comparison of Different Loss Functions

Five bounding box regression loss functions were investigated to assess the effects of various loss functions on detection accuracy: GIoU [46], Shape-IoU [40], Inner-IoU [47], WIoU [41], and the newly proposed DN-ShapeIoU. These were compared alongside the original CIoU loss function [44] used in YOLOv8n.

Table 6 presents the performance metrics for YOLOv8n using various bounding box regression loss functions. It is important to note that the model’s GFLOPs and parameter count were not significantly affected by these loss functions, and as a result, these metrics are not listed in the table. For the original YOLOv8n model with the CIoU loss function, mAP50, mAP95, R, and P are 93.6%, 54.1%, 90.1%, and 93.3%, respectively. The application of the GIoU loss function led to slight reductions in mAP50, mAP95, and R. Improvements in mAP50, mAP95, and R were obtained by switching to Inner-IoU and Shape-IoU loss functions; however, precision suffered decreases of 0.6% and 1.1%, respectively. This drop in precision could be attributed to the addition of auxiliary bounding boxes with Inner-IoU and the focus on bounding box dimensions and shapes with Shape-IoU, which enhance detection capabilities for small targets but may reduce precision. The WIoU v3 loss function showed improvements in all four metrics, but the model using DN-ShapeIoU achieved the highest performance. With DN-ShapeIoU, mAP50, mAP95, recall, and precision increased to 94.2%, 55.1%, 91.0%, and 94.0%, respectively. Compared to the original YOLOv8n, these improvements are 0.7% in mAP50, 1.0% in mAP95, 0.9% in recall, and 1.7% in precision. The improved performance of DN-ShapeIoU may be explained by its thorough evaluation of bounding box scale and shape in addition to sample quality. By using this technique, the network can process information from more difficult images, leading to better detection results.

### 3.4. Ablation Studies

Several ablation studies were carried out under controlled conditions to confirm the effectiveness of the enhancements. The evaluation metrics used included mAP50, mAP95, R, P, GFLOPs, Param, FPS, and model size. These metrics comprehensively assessed the performance of the models. The enhancements evaluated included the following: integrating depthwise separable convolutions in the backbone (DS), employing the proposed RCELAN structure in place of the C2f module in the neck section (R), applying the BiFPN architecture in the neck (B), and employing DN-ShapeIoU for bounding box regression (DN). Table 7 summarizes the experimental results of YOLOv8n and its improved versions: YOLOv8n + DS, YOLOv8n + DS + R, YOLOv8n + DS + R + B, and YOLOv8n + DS + R + B + DN (proposed DRBD-YOLOv8).

In comparison to the baseline YOLOv8n, the model that replaces standard convolutions in the backbone with depthwise separable convolutions (referred to as YOLOv8n + DS) achieved a 1% increase in mAP50. Moreover, GFLOPs, parameters, and model size were reduced by approximately 11%, demonstrating that depthwise separable convolutions improved detection accuracy while decreasing model complexity and computational requirements.

Substituting the C2F module with the RCELAN module in the neck network (referred to as YOLOv8n + DS + R) led to a slight decline in mAP50 and mAP95, though these changes are acceptable. GFLOPs and model size were reduced to 6.4 and 4.58 M, respectively, making the model more compact and lightweight. Recall and precision in particular increased significantly, which resulted in a decrease in miss and false alarm rates. The FPS rose from 340 to 357, demonstrating improved inference efficiency and speed.

The integration of the BiFPN architecture (referred to as YOLOv8n + DS + R + B) further improved mAP50 and mAP95 by approximately 0.5% compared to YOLOv8n + DS + R. BiFPN enhanced feature fusion across different levels, improving detection performance for small objects. This change shrunk the model size to a more compact 3.25 MB, and GFLOPs and parameters decreased by 11% and 30.8%, respectively. This suggests that the BiFPN architecture significantly lowers memory usage and computational costs, satisfying the needs of resource-constrained edge devices.

The application of DN-ShapeIoU as the loss function (referred to as YOLOv8n + DS + R + B + DN) achieved the highest performance with mAP50 and mAP95 reaching 95.1% and 55.4%, respectively. The reason for this improvement is that DN-ShapeIoU can handle low-quality samples more effectively, which increases training precision. The precision reduced slightly, while the recall climbed to 92%. Higher recall is essential for drone detection since it minimizes missed detections.

**Figure 8:**
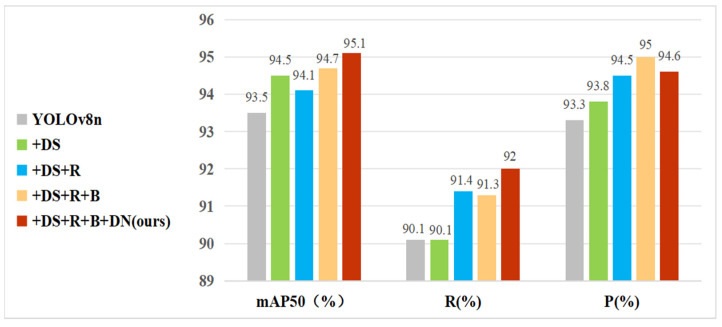
Comparative analysis of mAP50, recall, and precision.

**Figure 9:**
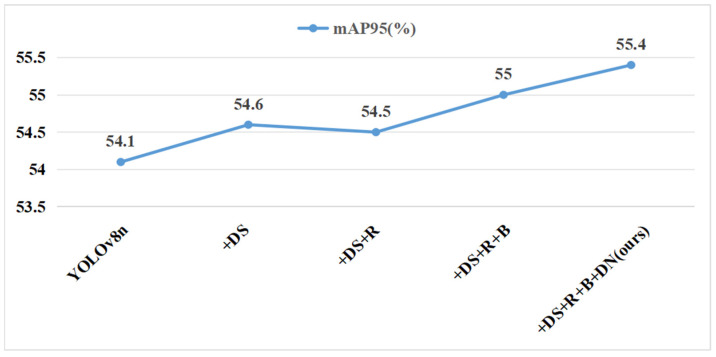
Comparative analysis of mAP95.

**Figure 10:**
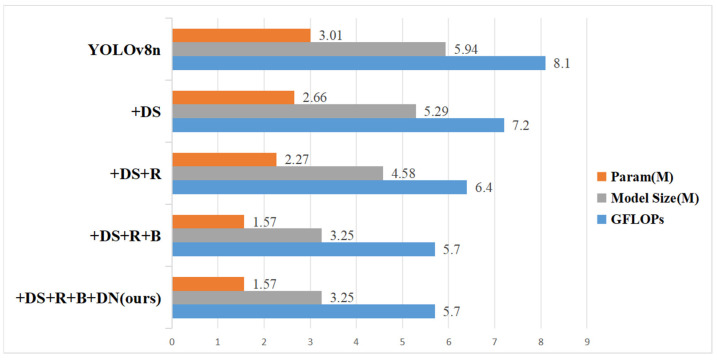
Comparative analysis of parameters, model size, and GFLOPs.

The DRBD-YOLOv8 model exceeds all other models in metrics such as mAP50, mAP95, recall, and FPS, while also having the smallest model size and lowest computational complexity (Figure 8, Figure 9 and Figure 10). Overall, the DRBD-YOLOv8 algorithm demonstrates superior performance.

### 3.5. Comparison of Different Object Detection Models

The proposed DRBD-YOLOv8 model was thoroughly compared with a series of object detection models to validate its efficiency and superiority. The comparison focused on several key evaluation metrics, including mAP50, P, GFLOPs, FPS, and model size.

Table 8 shows that the mAP50 for the proposed DRBD-YOLOv8 model is 95.1%, which is slightly lower than YOLOv5s (96.2%) and YOLOv8s (96.4%). However, DRBD-YOLOv8 achieves a model size of just 3.25 M and the highest FPS of 357 tasks per second. Compared to YOLOv7-tiny, YOLOv8n, YOLOv9t, and YOLOv10n, the DRBD-YOLOv8 model demonstrates better performance metrics. Although YOLOv5n has the lowest GFLOPs, its mAP50, precision, FPS, and model size are all less favorable than those of DRBD-YOLOv8. Specifically, YOLOv5n’s mAP50 and precision are 2.6% and 3.2% lower than DRBD-YOLOv8’s, respectively.

**Figure 11:**
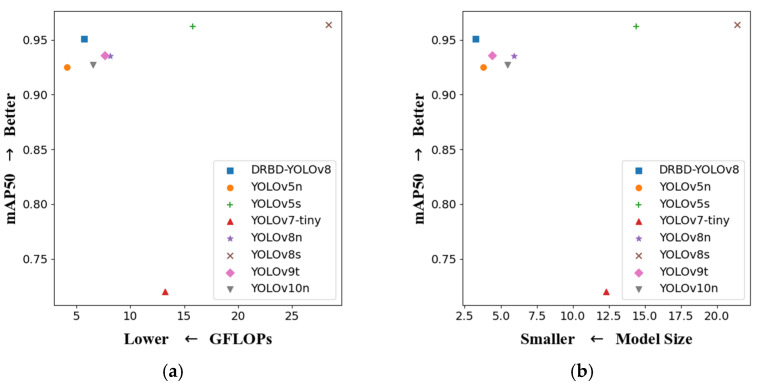

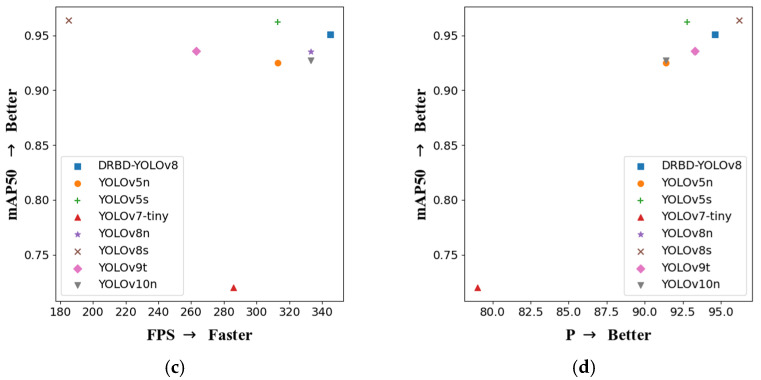
Comparison of different models for different indicators: (**a**) GFLOPs and mAP50, (**b**) model size and mAP50, (**c**) FPS and mAP50, and (**d**) P and mAP50.

For a more intuitive comparison of the comprehensive performance of DRBD-YOLOv8, mAP50 is plotted on the vertical axis, with four other evaluation metrics on the horizontal axis. Figure 11a,b display scatter plots of mAP50 versus GFLOPs and mAP50 versus model size, respectively. In these plots, points that are closer to the top-left corner indicate better performance. Figure 11c,d show scatter plots of mAP50 versus FPS and mAP50 versus precision, respectively, with points closer to the bottom-right corner signifying superior performance. The DRBD-YOLOv8 model is better overall. It provides the highest detection accuracy and FPS while maintaining the lowest computational complexity and smallest model size.

### 3.6. Experimental Results on the Public Dataset

The DUT anti-UAV dataset was employed to retrain, validate, and test the DRBD-YOLOv8 model to assess its robustness and generalization [49]. This dataset consists of 10,000 drone images, divided into 5200 for training, 2600 for validation, and 2200 for testing. It features over 35 drone models in a variety of scenes, lighting conditions, and weather scenarios, making it an ideal benchmark for evaluating the robustness and performance of drone detection models. The results of the comparative experiments indicate that DRBD-YOLOv8 outperforms YOLOv8n by 24 FPS, with improvements of 2.6% in mAP50, 1.1% in mAP95, 0.8% in recall, and 0.9% in precision (Table 9). In comparison to YOLOv8n, DRBD-YOLOv8 demonstrates superior performance across all evaluated metrics on the DUT anti-UAV dataset. These findings validate the outstanding generalization and robustness of the DRBD-YOLOv8 model.

### 3.7. Visualization Experiments

**Figure 12:**
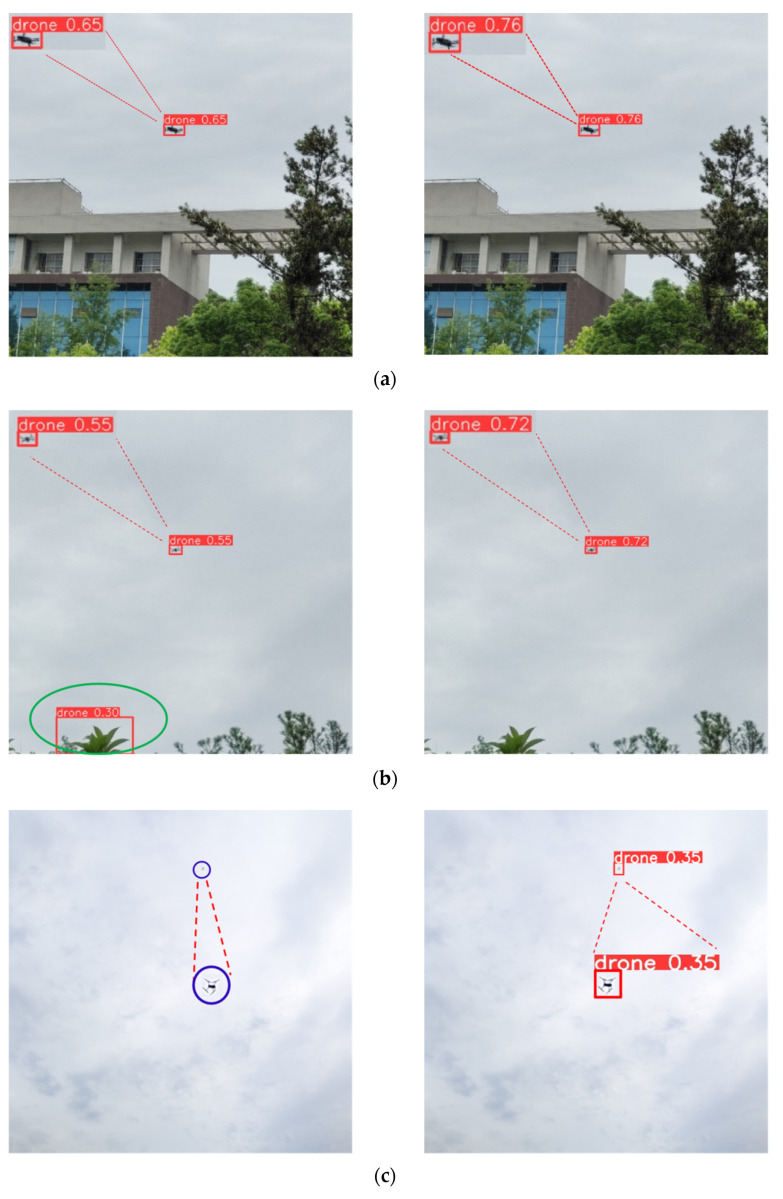

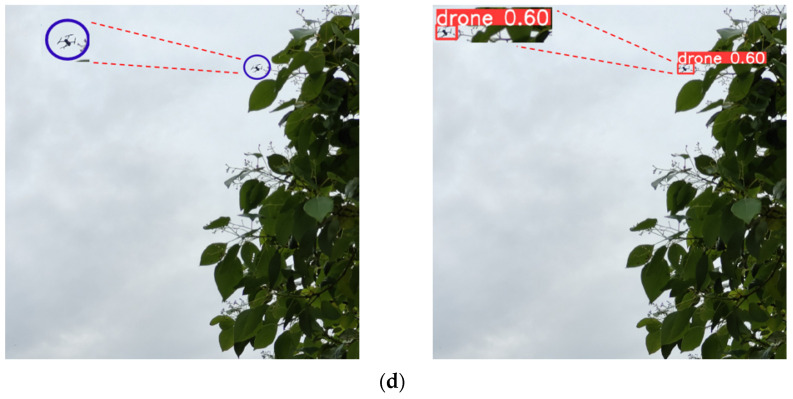
The detection results of YOLOv8n ((**left**) panel) and DRBD-YOLOv8 ((**right**) panel). (**a**) over urban areas. In (**b**), the circled green ellipse is a false detection object. In (**c**,**d**), those circled deep purple ellipses are missed detection objects.

Drone images from various scenarios were analyzed to compare the detection performance of DRBD-YOLOv8 and YOLOv8n. It is evident from Figure 12 that DRBD-YOLOv8 consistently outperforms the baseline model in detection accuracy, capability to identify small objects, and robustness to interference. Figure 12 enlarges the detection findings to provide a more readable representation. In Figure 12a, DRBD-YOLOv8 displays higher detection confidence on the same image compared to YOLOv8n. In Figure 12b, leaves are incorrectly classified as a drone by YOLOv8n, but the item is correctly classified as a drone by DRBD-YOLOv8. For very small targets, as shown in Figure 12c, YOLOv8n fails to detect a distant drone that occupies only a few pixels, while DRBD-YOLOv8 successfully identifies it. Moreover, Figure 12d depicts a situation where a drone flying at a high altitude is partially obscured by branches. YOLOv8n struggles to discern the drone due to the complex background, but DRBD-YOLOv8 accurately detects it.

Overall, DRBD-YOLOv8 successfully addresses the issues with false and missing detections that YOLOv8n introduced. The model is more sensitive to distant drones due to improvements in cross-layer feature fusion and multi-scale feature extraction. Further, to improve the robustness and performance of the developed model in challenging detection environments, the DN-ShapeIoU loss function adjusts learning gradients based on sample quality.

## 4. Discussion

The DRBD-YOLOv8 model incorporates several significant improvements compared to YOLOv8n. First, depthwise separable convolutions are added to the backbone network to improve detection performance while maintaining computational efficiency. Second, cross-scale information extraction and fusion are improved by the use of RCELAN modules and BiFPN architecture. These modifications improve the sensitivity of the model to small targets while significantly reducing its computing load and complexity. Last, the DN-ShapeIoU loss function produces better bounding box regression and overall detection accuracy because of its dynamic gradient modification dependent on sample quality.

DRBD-YOLOv8 demonstrates significant improvements over the original YOLOv8n, with a 1.6% increase in mAP50, a 1.3% enhancement in mAP95, a 1.9% higher recall rate, and a 1.3% higher precision rate. Moreover, the GFLOPs and parameters of the model are reduced by 29.6% and 47.8%, respectively, achieving a compact size of 3.25 M, nearly halving that of the original model. DRBD-YOLOv8 also achieves a high FPS of 357, fulfilling real-time detection requirements. The robust performance of the developed model on the public DUT anti-UAV dataset further confirms its generalization and reliability. Comparative experiments with other leading detection models highlight the advantages of DRBD-YOLOv8 in lightweight design, detection speed, and accuracy, making it ideal for deployment in vehicle security systems, mobile devices, and edge-computing devices for real-time drone detection and airspace threat monitoring.

However, DRBD-YOLOv8 possesses some limitations. Although there have been improvements in detecting speed with DRBD-YOLOv8, the degree of this increase is still quite small. To further improve detection speed, future studies might concentrate on investigating model compression strategies. Furthermore, the model does not take into consideration the effect of weather on detection performance. Sophisticated algorithms for rain removal and image defogging need to be integrated to improve the model’s performance under adverse weather conditions, which would increase its generalization potential. In addition, future research will also involve deploying the model on edge-computing devices, conducting experiments for real UAV detection, and fine-tuning the model based on the experimental results.

## 5. Conclusions

This study presents DRBD-YOLOv8, an optimized drone detection model that incorporates several significant improvements compared to YOLOv8n. These include the incorporation of depthwise separable convolutions in the backbone, the use of an RCELAN fusion module and BiFPN structure in the neck, and the application of the DN-ShapeIoU loss function. DRBD-YOLOv8 was designed for high-speed computation, increased accuracy, reduced computational requirements, and low memory usage, making it an ideal choice for real-time detection on embedded edge devices.

## Figures and Tables

**Table 1:** The key training parameter configurations.

Parameters	Setup
Epochs	200
Initial learning rate	0.01
Image size	640 × 640
Batch size	32
Optimizer	SGD
Momentum	0.937
Weight decay	0.0005
NMS IOU	0.7
Mosaic	1.0
Close mosaic	Last 10 epochs

**Table 2:** Comparison experiment of different convolutions.

Convolutions	mAP50 (%)	mAP95 (%)	R (%)	P (%)	GFLOPs	Param (M)
Conv	93.5	54.1	90.1	93.3	8.1	3.01
DWConv	93.6	54.8	90.1	93.2	7.2	2.62
GhostConv	93.0	53.3	90.0	93.9	7.6	2.82
PWConv	92.2	51.5	89.7	92.2	7.2	2.66
GConv	94.1	54.8	90.2	93.5	7.3	2.66
DSConv	94.5	55.1	90.1	93.8	7.2	2.66

**Table 3:** A comparison of different feature extraction modules.

Module	Param (kB)	GFLOPs	FPS	Network
C3	295.7	4.8	522.1	YOLOv5
ELAN	492.3	8.1	391.0	YOLOV7
C2f	459.5	7.5	388.2	YOLOv8
RepNCSPELAN4	226.2	3.7	527.9	YOLOv9
RCELAN	209.5	3.4	691.6	DRBD-YOLOv8 (ours)

**Table 4:** Comparison experiments with RCELAN module replacement of C2f modules at different positions.

Model	Groups	Replacement Position	mAP50 (%)	mAP95 (%)	R (%)	P (%)	GFLOPs	Param (M)
YOLOv8n	1	-	93.5	54.1	90.1	93.3	8.1	3.01
	2	Backbone	93.5	53.8	90.1	93.1	7.4	2.79
	3	Neck	93.8	54.8	89.9	94.1	7.3	2.62
	4	All	93.1	53	89.7	93.0	6.6	2.40

**Table 5:** Comparison experiments with BiFPN.

Model	mAP50 (%)	mAP95 (%)	R (%)	P (%)	GFLOPs	Param (M)	Model Size (M)
YOLOv8n	93.5	54.1	90.1	93.3	8.1	3.01	5.94
YOLOv8n + BiFPN	93.9	54.8	90.4	94.1	7.1	1.99	4.02

**Table 6:** Comparative experiments with different loss functions.

Model	Loss Function	mAP50 (%)	mAP95 (%)	R (%)	P (%)
YOLOv8n	CIoU	93.5	54.1	90.1	93.3
	GIoU	93.4	53.8	89.4	93.7
	Inner-IoU (ratio = 1.05)	94.0	54.7	90.9	92.7
	Shape-IoU	94.2	54.5	91.0	92.2
	WIoU v3	94.1	54.8	90.8	93.5
	DN-ShapeIoU	94.2	55.1	91.0	94.0

**Table 7:** Results of ablation studies.

Model	mAP50 (%)	mAP95 (%)	R (%)	P (%)	GFLOPs	Param (M)	FPS	Model Size (M)
YOLOv8n	93.5	54.1	90.1	93.3	8.1	3.01	333	5.94
+ DS	94.5	54.6	90.1	93.8	7.2	2.66	340	5.29
+ DS + R	94.1	54.5	91.4	94.5	6.4	2.27	357	4.58
+ DS + R + B	94.7	55.0	91.3	95	5.7	1.57	357	3.25
+ DS + R + B + DN (ours)	95.1	55.4	92	94.6	5.7	1.57	357	3.25

**Table 8:** Experimental results of other mainstream object detection models.

Model	mAP50 (%)	P (%)	GFLOPs	FPS	Model Size (M)
YOLOv5n [42]	92.5	91.4	4.1	313	3.8
YOLOv5s [42]	96.2	92.8	15.8	313	14.4
YOLOv7-tiny [43]	72	79	13.2	286	12.3
YOLOv8n [44]	93.5	93.3	8.1	333	5.94
YOLOv8s [44]	96.4	96.2	28.4	185	21.4
YOLOv9t [45]	93.6	93.3	7.6	263	4.42
YOLOv10n [48]	92.7	91.4	6.5	333	5.48
DRBD-YOLOv8 (ours)	95.1	94.6	5.7	357	3.25

**Table 9:** Comparative experiments with the DUT anti-UAV dataset.

Model	mAP50 (%)	mAP95 (%)	R (%)	P (%)	FPS
YOLOv8n	83.1	52.4	73.5	90.1	333
DRBD-YOLOv8 (ours)	85.7	53.5	77	90.9	357

## Data Availability

The data presented in this study are available upon request from the corresponding author due to privacy reasons.
